# Model for End-stage Liver Disease excluding INR (MELD-XI) score in critically ill patients: Easily available and of prognostic relevance

**DOI:** 10.1371/journal.pone.0170987

**Published:** 2017-02-02

**Authors:** Bernhard Wernly, Michael Lichtenauer, Marcus Franz, Bjoern Kabisch, Johanna Muessig, Maryna Masyuk, Uta C. Hoppe, Malte Kelm, Christian Jung

**Affiliations:** 1 Clinic of Internal Medicine II, Department of Cardiology, Paracelsus Medical University of Salzburg, Salzburg Austria; 2 Clinic of Internal Medicine I, Department of Cardiology, Jena University Hospital, Jena, Germany; 3 Division of Cardiology, Pulmonology, and Vascular Medicine, Medical Faculty, University Duesseldorf, Duesseldorf, Germany; Columbia University, UNITED STATES

## Abstract

**Purpose:**

MELD-XI, an adapted version of Model for End-stage Liver Disease (MELD) score excluding INR, was reported to predict outcomes e.g. in patients with acute heart failure. We aimed to evaluate MELD-XI in critically ill patients admitted to an intensive care unit (ICU) for prognostic relevance.

**Methods:**

A total of 4381 medical patients (66±14 years, 2862 male) admitted to a German ICU between 2004 and 2009 were included and retrospectively investigated. Admission diagnoses were e.g. myocardial infarction (n = 2034), sepsis (n = 694) and heart failure (n = 688). We divided our patients in two cohorts basing on their MELD-XI score and evaluated the MELD-XI score for its prognostic relevance regarding short-term and long-term survival. Optimal cut-offs were calculated by means of the Youden-Index.

**Results:**

Patients with a MELD-XI score >12 had pronounced laboratory signs of organ failure and more comorbidities.

MELD-XI >12 was associated with an increase in short-term (27% vs 6%; HR 4.82, 95%CI 3.93–5.93; p<0.001) and long-term (HR 3.69, 95%CI 3.20–4.25; p<0.001) mortality. In a univariate Cox regression analysis for all patients MELD-XI was associated with increased long-term mortality (changes per score point: HR 1.06, 95%CI 1.05–1.07; p<0.001) and remained to be associated with increased mortality after correction in a multivariate regression analysis for renal failure, liver failure, lactate concentration, blood glucose concentration, oxygenation and white blood count (HR 1.04, 95%CI 1.03–1.06; p<0.001). Optimal cut-off for the overall cohort was 11 and varied remarkably depending on the admission diagnosis: myocardial infarction (9), pulmonary embolism (9), cardiopulmonary resuscitation (17) and pneumonia (17). We performed ROC-analysis and compared the AUC: SAPS2 (0.78, 95%CI 0.76–0.80; p<0.0001) and APACHE (0.76, 95%CI 0.74–0.78; p<0.003) score were superior to MELD-XI (0.71, 95%CI 0.68–0.73) for prediction of mortality.

**Conclusions:**

The easily calculable MELD-XI score is a robust and reliable tool to predict both intra-ICU and long-term mortality in critically ill medical patients admitted to an ICU. Optimal cut-off values for MELD-XI scores seem to depend on the primary disease and need to be validated in future prospective studies. Compared to SAPS2 and APACHE score, MELD-XI lacks precision but might have comparable and even additive value, as it is easily available and independent of subjective values.

## Introduction

Patients admitted to an intensive care unit (ICU) represent a highly heterogeneous population. They largely differ in terms of clinical presentation, age, disease etiology, hemodynamics, treatment response as well as in prognosis. Scoring systems (such as APACHE 2 and SAPS2) have been developed to better stratify the risk profiles of ICU patients and to estimate their potential outcome [1–3].

In a plethora of studies, the utility of the Model for End-Stage Liver Disease (MELD) score has been evaluated as a predictor for clinical outcome in patients suffering from liver disease. It utilizes a logarithmic function including serum creatinine, total serum bilirubin and the International Normalized Ratio (INR) [4]. It has been shown that the MELD score can serve as an indicator of multi-organ failure [5]. The score is currently used in the allocation of organs for patients waiting for liver transplantation as it also correlates significantly with waiting list mortality [6]. The MELD score captures derangements in two critical organ systems: kidney and liver. Liver dysfunction and elevations in the associated serum markers are known to be related to poor outcomes in many patient collectives [7]. Increased serum total bilirubin is known to be linked with hepatocellular hypoxia originating from low cardiac output and/or increased hepatic venous pressure [8–10]. Recently liver function has been reported to predict survival in surgical patients undergoing extracorporal membrane oxygenation, and hepato-cardiac comorbidities have come into the spotlight for risk stratification of those critically ill patients [11, 12]. As the kidney is depending on constant and adequate blood flow it effectively mirrors states of global hypoperfusion and venous congestion, conditions which are common in critically ill patients [13].

As it is including INR in the equation MELD score cannot be applied for patients being on treatment with oral anticoagulants such as warfarin or phenprocoumon which is a major limitation in medical patients. Therefore, a modification of the MELD score excluding INR (MELD-XI score) was designed and it could be shown that the MELD-XI score, even though it omitted INR from the equation, is comparable to the MELD score with respect to accurateness in predicting mortality in patients suffering from liver cirrhosis [14].

Critically ill patients treated in an intensive care unit represent a heterogeneous collective. They differ decisively in their clinical presentation, age, etiology and pathophysiology of the underlying disease and preexisting medical conditions, inflammatory state, medication and hemodynamics. It is of utmost importance to identify patients at highest risk at an early time point, ideally directly at initial admission at the ICU, to intensify therapeutic strategies and eventually extend monitoring to optimize outcome for those patients. On the other hand, in an aging population and an estimated increase of e.g. heart failure prevalence of about 25% until 2030 compared to 2013, it is of equal interest sto have reliable scores to determine which patients are at low risk for death to focus intensive care treatment on those patients who really depend on and could profit from it [15–19].

Today, there are a variety of scores for risk stratification, with Acute Physiology And Chronic Health Evaluation (APACHE) and Simplified Acute Physiology Score II (SAPS2) being the most widely used [3, 20]. Whereas those scores are well validated and comprehensive, they are difficult to calculate and depend on information regarding pre-existing illnesses, which might not always be known at ICU admission due to the emergency setting, or parameters, which are highly variable such as breathing frequency, heart rate and body temperature.

Motivated by these considerations, in the current study we aimed to evaluate MELD-XI in a large collective of critically ill patients admitted to an intensive care unit with respect to prognostic relevance regarding (i) short-term and (ii) long-term mortality. Moreover, we (iii) compared it to existing well-accepted scores (APACHE, SAPS2) and calculated optimal cut-off values.

## Methods

### Study subjects

4688 patients that were treated at an ICU at the University Hospital Jena between January 2004 and December 2009 were enrolled in this study. Inclusion criteria was admission to the medical ICU at University Hospital Jena, there were no exclusion criteria. For baseline characteristics 4381 patients were investigated, as for the other 218 patients lab values were not recorded date specific. Standard laboratory values, patient`s medical history and clinical data were documented. All laboratory values were obtained from standard in-hospital laboratory. Follow-up of patients was performed retrospectively between May 2013 and November 2013. The primary endpoint of the study was all cause mortality. Mortality data were collected by review of medical records in our COPRA patient data management system (COPRA System GmbH, Berlin, Germany) and/or patient contact. The study has been approved by the local ethics committee of the Jena University Hospital.

### Statistical analysis

Normally distributed data points are expressed as mean ± standard deviation. Differences between independent groups were calculated using the Student’s t-test. Non-normally distributed continuous variables are expressed as medians (interquartile range) and compared using Mann-Whitney U test. Categorical data are expressed as numbers (percentage). Chi-square test was applied to calculate differences between groups. Survival rates were calculated using chi-square test for analysis of intra-ICU mortality and both univariate and multivariate Cox regression analysis to adjust for cofounding factors for long-term mortality. For the multivariate regression model, cofounders with a p-value <0.10 in the univariate analysis were included, then a backward variable elimination was performed. Elimination criterion was a p-value of more than 0.10. A p-value of <0.05 was considered statistically significant. We report several multivariate regression models for separate organ systems and one integrated model which adjusts for several cofounders. SPSS version 22.0 (IBM, USA) and MedCalc version 14.8 (MedCalc Software, USA) were used for all statistical analyses.

### Calculation of MELD-XI, SAPS2 and APACHE score

MELD-XI score was calculated as follows: MELD-XI = 5.11 x ln (serum bilirubin in mg/dL) ± 11.76 x ln (serum creatinine in mg/dL) ± 9.44. Serum creatinine and bilirubin values utilized were those reported on day of admission. If there was more than one value on admission day, the highest was utilized. Initial Simplified Acute Physiology Score II (SAPS2) and Acute Physiology And Chronic Health Evaluation (APACHE) scores were calculated by the treating physician within 24 hours after admission as reported before [3, 20].

## Results

### Study population

4381 patients were included in our retrospective analysis. Most of our patients were admitted for myocardial infarction (n = 2034), sepsis (n = 694), heart rhythm disturbance (846) and heart failure (n = 688). Of note, in some patients was more than one primary admission diagnosis reported, e.g. both myocardial infarction and heart failure. Due to missing values we could calculate MELD-XI score only for 3637 patients. The median MELD-XI score was 9.5 (IQR 5.5–15.7). After review of available literature, we decided to split our study population in two cohorts, representing high versus low MELD-XI score patients: MELD-XI > 12 (n = 1434) and < 12 (n = 2203). Baseline characteristics of the study population are presented in Tables 1 and 2. Patients with a MELD-XI score > 12 were older (68 ± 13 years vs 65 ± 14 years; p<0.001), had higher lactate levels at admission (2.6 ± 4.2 mmol/L vs 2.0 ± 2.8 mmol/L; p<0.001) and had more pronounced laboratory signs of organ failure: creatinine was increased (266.8 ± 193.5 μmol/L vs 90.4 ± 26.4 μmol/L; p<0.001), liver parameters such as transaminases (alanine-aminotransferase (ALAT) 4.4 ± 12.1 μmol/l*s vs 1.1 ± 2.6 μmol/l*s; p<0.001; aspartate aminotransferase (ASAT) 9.0 ± 28.5 μmol/l*s vs 2.4 ± 4.8 μmol/l*s; p<0.001) and Gamma-GT (2.2 ± 2.7 μmol/l*s vs 1.4 ± 2.2 μmol/l*s; p<0.001) were significantly higher. Additionally, these patients with a MELD-XI score > 12 showed a more severe hypoxemic respiratory insufficiency represented by lower pO2 (8.9 ± 1.9 kPa vs 9.1 ± 2.1 kPa; p<0.001) and had lower hemoglobin concentrations (6.7 ± 1.2 mmol/L vs 7.6 ± 1.2 mmol/L; p<0.001). Patients with a high MELD-XI score had significantly higher APACHE (26 ± 9 vs 18 ± 9; p<0.001) and SAPS2 (52 ± 19 vs 36 ± 18; p<0.001) scores.

**Table 1:** Baseline characteristics of the study population. Patients with a MELD-XI score above 12 were older (68 ± 13 years vs 65 ± 14 years; p<0.001), had higher lactate levels at admission (2.6 /L ± 4.2 mmol/L vs 2.0 ± 2.8 mmol/L; p<0.001) and had more pronounced laboratory signs of organ failure. Normally distributed data points are expressed as mean ± standard deviation.

	MELD >12	MELD <12	p =
creatinin (μmol/l)	266.80 (±193.50)	90.42 (±26.44)	<0.001
urea (μmol/l)	20.11 (±12.62)	6.99 (±4.16)	<0.001
bilirubin (μmol/l)	33.93 (±54.98)	13.78 (±7.68)	<0.001
cholinesterase (μmol/l)	64.41 (±34.98)	99.81 (±41.95)	<0.001
gamma GT (μmol/l*s)	2.19 (±2.70)	1.36 (±2.17)	<0.001
GLDH (μmol/l*s)	4064.23 (±17113.51)	389.66 (±2224.01)	<0.001
ALAT (μmol/l*s)	4.35 (±12.09)	1.08 (±2.58)	<0.001
ASAT (μmol/l*s)	9.00 (±28.50)	2.40 (±4.81)	<0.001
pCO2 (kPa)	6.38 (±2.02)	6.26 (±2.07)	0.305
pO2 (kPa)	8.85 (±1.87)	9.11 (±2.05)	<0.001
haemoblobine (mmol/l)	6.74 (±1.15)	7.55 (±1.21)	<0.001
lactate (mmol/l)	2.60 (±4.18)	2.01 (±2.80)	<0.001
BMI	27.53 (±5.16)	27.54 (±5.28)	0.981
age (years)	68 (±13)	65 (±14)	<0.001

**Table 2:** A high MELD-XI score identified sicker patients with multiple preconditions. Normally distributed data points are expressed as mean ± standard deviation.

	MELD >12	MELD <12	p =
renal insufficiency	2%	0%	<0.001
type 2 diabetes mellitus	17%	12%	<0.001
dementia	2%	2%	n.s.
mitral valve insufficiency	4%	3%	n.s.
aortic valve stenosis	4%	3%	n.s.
CVD	26%	43%	<0.001
one vessel disease	6%	13%	<0.001
two vessel disease	7%	13%	<0.001
three vessel disease	13%	16%	n.s.
history of atrial fibrillation	21%	11%	<0.001
chronic heart failure	17%	10%	<0.001
NYHA III	5%	3%	0.001
NYHA IV	11%	6%	<0.001
history of stroke	2%	1%	n.s.
peripheral artery disease	2%	1%	<0.001
COPD	7%	6%	n.s.
ASH	4%	1%	<0.001
NASH	3%	1%	<0.001
arterial hypertension	20%	30%	<0.001
APACHE2 Score	18 (±9)	26 (±9)	<0.001
SAPS2 Score	36 (±18)	52 (±19)	<0.001

Regarding pre-existing diseases, a high MELD-XI score identified patients with multiple preconditions (Table 2): The high MELD-XI cohort included more diabetics (17% vs 12%; p<0.001), more chronic heart failure patients (17 vs 10%; p<0.001) and more patients with atrial fibrillation (21% vs 11%; p<0.001). However, patients with low MELD-XI scores showed a higher prevalence of coronary artery disease (26% vs 43%; p<0.001) in general but there were no differences regarding the presence of three-vessel-disease (16% vs 13%, p = 0.4).

### Survival data

The high MELD-XI cohort experienced significantly higher intra-ICU mortality (27% vs 6%; HR 4.82, 95%CI 3.93–5.93; p<0.001) for the overall patient collective. An admission MELD-XI score above 12 was associated with worse intra-ICU outcome for all sub-populations at a cut-off value of 12, e.g. pneumonia (HR 2.15; 95%CI 1.38–3.35; p = 0.001), acute myocardial infarction (HR 7.10, 95%CI 4.57–11.02; p<0.001), sepsis (HR 2.40; 95%CI 1.55–3.72, p<0.001) which is shown in Table 3.

**Table 3:** A MELD-XI >12 predicted increased intra-ICU mortality regardless of primary/secondary diagnosis.

admission diagnosis	HR	95%CI	p =	n =	intra-ICU mortality(%). MELD > 12	intra-ICU mortality(%). MELD < 12
overall cohort	4.82	3.93–5.93	<0.001	3234	27%	6%
pneumonia	2.15	1.38–3.35	0.001	466	31%	17%
myocardial infarction	7.10	4.57–11.02	<0.001	1557	18%	3%
sepsis	2.40	1.55–3.72	<0.001	487	45%	26%
pulmonary embolism	5.59	1.95–9.49	<0.001	122	44%	12%
acute heart failure	2.37	1.46–3.83	<0.001	499	25%	13%
cardiopulmonary reanimation	2.77	1.71–4.48	<0.001	360	39%	19%

**Fig 1:**
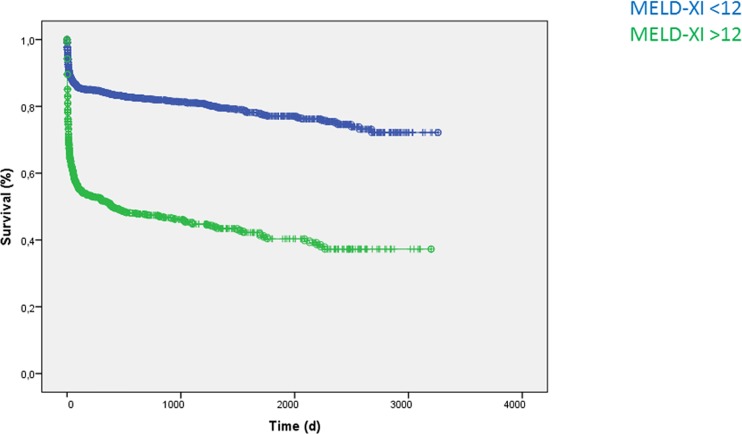
Patients with a MELD-XI >12 at admission showed significantly increased long-term mortality (HR 3.69, 95%CI 3.20–4.25; p<0.001).

Additionally, patients with a MELD-XI >12 at admission had a significantly increased long-term mortality (HR 3.69, 95%CI 3.20–4.25; p<0.001; Fig 1). In a univariate Cox regression analysis MELD-XI was associated with increased long-term mortality (changes per score point: HR 1.06, 95%CI 1.05–1.07; p<0.001). MELD-XI was as well associated with worse long-term outcome regardless for all sub-populations, e.g. pneumonie (changes per score point: HR 1.02; 95%CI 1.01–1.04; 0.007), acute myocardial infarction (changes per score point: HR 1.10; 95%CI 1.09–1.12, p<0.001), sepsis (HR 1.02, 95%CI 1.01–1.04; p = 0.001) as seen in Table 4.

**Table 4:** In a Cox regression analysis MELD-XI (changes per unit in points) was associated with increased long-term mortality regardless of admission diagnosis.

admission diagnosis	HR	95%CI	p =	n =	optimal cut-off	median MELD-XI
overall cohort	1.06	1.05–1.07	<0.001	3140	11	10
pneumonia	1.02	1.01–1.04	0.007	444	17	12
myocardial infarction	1.10	1.09–1.12	<0.001	1532	9	7
sepsis	1.02	1.01–1.04	0.001	463	15	17
pulmonary embolism	1.11	1.07–1.15	<0.001	117	9	7
heart failure	1.04	1.02–1.05	<0.001	472	15	13
cardiopulmonary reanimation	1.06	1.04–1.08	<0.001	352	17	12
heart rhythm distrubance	1.08	1.06–1.10	<0.001	580	15	12

Further multivariate regression analysis was done: After correction for parameters of liver failure (ALAT, ASAT and bilirubin) alone MELD-XI remained associated with increased mortality (changes per score point: HR 1.05, 95%CI 1.04–1.06; p<0.001). Likewise, after correction for parameters of renal failure (creatinine, urea, potassium) alone MELD-XI remained associated with adverse outcome (HR 1.09, 95%CI 1.07–1.11; p<0.001; n = 2531). After correction for both liver (ALAT, ASAT, bilirubin) and renal (creatinine, urea, potassium) failure, as well as lactate concentration as marker of tissue under-perfusion, both glucose concentration as well as white blood count as well-known stress parameters, and further pO2 as marker of oxygenation, MELD-XI was still associated with mortality (HR 1.04 95%CI 1.03–1.06; p<0.001; Table 5). S1 Table shows all relevant data regarding survival and MELD-XI.

**Table 5:** MELD-XI was still associated with mortality (HR 1.04 95%CI 1.03–1.06; p<0.001 in an adjusted model after correction for relevant cofounders.

	univariate HR (95%CI)	p-value	multivariate HR (95%CI)	p-value
MELD-XI	1.06 (1.05–1.07)	<0.001	1.04 (1.03–1.06)	<0.001
creatinine (μmol/L)	1.001 (1.001–1.002)	<0.001	0.998 (0.997–0.998)	<0.001
urea (μmol/L)	1.04 (1.03–1.04)	<0.001	1.03 (1.02–1.04)	<0.001
bilirubin (μmol/L)	1.005 (1.005–1.006)	<0.001	1.00 (0.99–1.002)	0.88
ALAT (μmol/L*s)	1.02 (1.017–1.029)	<0.001	0.99 (0.97–1.01)	0.11
ASAT (μmol/L*s)	1.011 (1.009–1.013)	<0.001	1.003 (1.000–1.006)	0.045
lactate (mmol/L)	1.06 (1.05–1.06)	<0.001	1.10 (1.09–1.12)	<0.001
glucose (mmol/L)	1.006 (1.002–1.009)	<0.001	1.001 (0.99–1.04)	0.93
leucocytes (G/L)	1.007 (1.005–1.010)	<0.001	1.008 (1.000–1.015)	0.053
potassium (mmol/L)	1.71 (1.51–1.93)	<0.001	1.06 (0.98–1.15)	0.13
pO2 (kPa)	0.895 (0.863–0.928)	<0.001	0.91 (0.87–0.96)	<0.001

### Optimal cut-off values depend on the underlying disease

Median MELD-XI was for the overall cohort, medians for subpopulations are given in Table 4. By means of the Youden-Index we calculated an optimal cut-off value for our study population. This MELD-XI score value was 11 and therewith only slightly lower compared to the value of 12 as calculated in previously published studies [21, 22]. Interestingly, an optimal cut-off varied remarkably depending on the admission diagnosis. Whereas for patients that underwent cardiopulmonary resuscitation and those admitted for pneumonia, the optimal cut-off value was a MELD-XI score of 17, and therewith higher than the empirical cut-off of 12 used by us, the optimal cut-off value for patients admitted for myocardial infarction and pulmonary embolism was 9, i.e. lower (Table 4).

### Comparison to SAPS2 and APACHE and other risk factors

SAPS2 and APACHE scores are widely used and well accepted for risk stratification and mortality prediction in critically ill patients. We therefore calculated AUC for APACHE score, SAPS2 score and age besides MELD-XI. SAPS2 score (0.78 95%CI 0.76–0.80; p<0.0001) and APACHE score (AUC 0.76, 95%CI 0.74–0.78; p<0.003) were superior to MELD-XI score (AUC 0.71, 95%CI 0.68–0.73) but age at admission (AUC 0.58, 95%CI 0.56–0.60; p<0.0001) was inferior to MELD-XI score for prediction of mortality (Table 6). Of note, liver transaminases, i.e. ALAT (AUC 0.55, 95%CI 0.52–0.58; p<0.001) and ASAT (AUC 0.55, 95%CI 0.52–0.57; p<0.001) were both inferior for predicting mortality than MELD-XI, which includes total serum bilirubin for assessment of liver dysfunction.

**Table 6:** Comparison of MELD-XI score to APACHE and SAPS2 scores: ROC—analysis was performed and AUC calculated.

	AUC (95%CI)	p = (vs MELD-XI)	HR	95%CI
SAPS2	0.78 (0.76–0.80)	<0.001	1.04	1.037–1.044
APACHE	0.76 (0.74–0.78)	0.003	1.08	1.072–1.088
MELD-XI	0.71 (0.68–0.73)		1.06	1.055–1.067
age	0.58 (0.56–0.60)	<0.001	1.02	1.019–1.027

S2 Table shows a STROBE checklist.

## Discussion

Risk stratification in critically ill patients admitted to an ICU is of crucial impact with respect to clinical management tending to improved patients’ outcomes as well as health economic aspects. There are few well-established scoring systems for individual risk assessment, e.g. the APACHE or the SAPS2 score. A major disadvantage of these scores is their complexity, which causes a limited feasibility in daily routine clinical practice. The MELD score and also the MELD-XI score, both assessing renal and liver dysfunction, have been originally developed and evaluated for the allocation of organs for patients waiting for liver transplantation. In addition, they could be proven to be appropriate for risk stratification also in other severe disorders like acute heart failure [23, 24]. Moreover, MELD-XI was reported to predict outcomes in patients undergoing heart transplantation as well as implantation of ventricular assist devices [25–27]. It was further shown that MELD-XI score provides additional risk stratification in patients suffering from acute heart failure by assessing renal and liver dysfunction which both are known to be associated with mortality in critically ill patients in general [24]. Abe et al. could show that MELD-XI predicts adverse prognosis in heart failure and high MELD-XI scores are associated with echocardiographic parameters of right ventricular overload such as increased right ventricular dilatation, increased inferior caval vein diameter as well as higher systolic pulmonary artery pressures [28]. A great advantage especially of the MELD-XI score is its simplicity enabling fast bedside risk stratification even in the emergency setting. Motivated by these considerations, the current study was designed to test the value of the MELD-XI score in ICU patients in a real-life setting. To our best knowledge, this is the first study in this vein and therefore of high clinical interest. As a result, patients with a high MELD-XI score were older, had significantly increased markers of multi-organ failure and had more pre-existing illnesses. The finding that patients with MELD<12 have a higher incidence of CVD most probably reflect that patients with myocardial infarction have lower MELD-XI scores. MELD-XI was significantly associated with both increased long-term and short-term (i.e. intra-ICU) mortality in our collective. This remained true even after correction for age, white blood count, oxygenation and lactate levels at admission in a multivariate cox regression analysis, parameters which are known to be associated with mortality [29–31]. This emphasizes how effective MELD-XI mirrors organ failure of two central organ systems: kidneys and liver, which are both sensitive for global hypo-perfusion and hypoxia [32, 33]. Interestingly, serum total bilirubin included in MELD-XI was superior to liver transaminases—which are frequently used to evaluate liver dysfunction—for prediction of mortality. This further supports the notion that increased serum total bilirubin is tightly associated with hepatocellular hypoxia [8–10].

The pathophysiologic mechanism that seems to be causative for the decline of liver function in (multi-) organ failure is thought to be related to hemodynamic disturbances within liver circulation. Elevated hepatic venous pressure coinciding with hypoxia and a decrease in blood flow leads to a subsequent loss of liver cells [8]. It has been shown previously that elevation of central venous pressure could contribute to cholestasis and a relevant reduction of hepatic function [34]. Not only obstruction of the biliary systems but also local hypoxia leads to an increase of serum bilirubin concentration, a pathophysiological phenomenon which has been described in many previous studies showing that bilirubin levels correlate with hemodynamic parameters, e.g. pulmonary artery wedge pressure and left ventricular function [34, 35]. Elevated bilirubin was reported to be associated with risk of pump failure in heart failure patients [36]. Further, diastolic dysfunction was associated with serum bilirubin levels in patients suffering from heart failure with preserved ejection fraction, bilirubin has been shown to predict mortality in chronic heart failure and pulmonary hypertension [37–40].

Optimal cut-offs to identify patients at increased risk for mortality differed considerably depending on primary admission diagnosis, indicating a value of MELD-XI in predicting outcome in both fulminant diseases like sepsis or after cardiopulmonary resuscitation—which come along with a pronounced increase of laboratory markers of organ failure—as well as subtler changes in renal and hepatic perfusion in diseases like myocardial infarction and pulmonary embolism.

Compared to APACHE and SAPS2 score, MELD-XI lacks both sensitivity and specificity in some degree for prediction of mortality. Nevertheless, against the background of the current findings, it can be postulated that the MELD-XI score might be of value for initial patient evaluation and risk stratification since it is much easier to calculate, does not depend on subjective and observer dependent clinical assessment (such as Glasgow Coma Scale) and gets along without volatile parameters often influenced by initial treatment (such as blood pressure, heart rate and oxygenation) which could make it more reproducible than both APACHE and SAPS2 score. Thus, this score might be easy to implement and seems to serve as a very robust bedside risk assessment tool especially in the emergency setting. As MELD-XI score bases on two parameters, which easily and cheaply can be measured by routine laboratory testing and further reflect two critical organ systems, there might even be a role for repetitive MELD-XI score calculation and an investigation of “MELD-XI clearance” in patients treated at an ICU over longer time periods. Furthermore, the MELD-XI score might be useful not only for mortality prediction but is known to correlate with future renal failure [22]. Risk stratification using MELD-XI score could therefore optimize patient`s long-term outcome and well-being by recommending intensive and more frequent follow-up for patients evidencing high MELD-XI scores during intensive care treatment. Thus, besides mortality prediction, MELD-XI score assessment might help to improve long-term quality of life in critically ill patients already at the very early time point of initial clinical presentation at the ICU.

### Limitations

Main limitations of our study are (i) its retrospective design and (ii) its single-center approach. Because of missing values, we could not include all our patients in survival and ROC analyses which could theoretically cause selection bias. In some patients, there was more than one primary admission diagnosis which (i) led to inclusion of those patients in calculation twice and (ii) therefore could limit precision of the disease-specific cut-offs we calculated. We think on the other hand that patients with multiple conditions, e.g. heart failure due to myocardial infarction, reflect a typical cohort of critically ill medical patients. Further, changing optimal cut-offs could be a sign of lacking precision of our score, which we cannot rule out completely.

### Conclusions

The MELD-XI score is a robust and reliable score to predict both short-term, i.e. intra-ICU, and long-term mortality in patients admitted to an ICU. Patients with higher MELD-XI score show significantly increased mortality even after correction for age and other clinically relevant parameters. Compared to SAPS2 and APACHE score, MELD-XI lacks precision but it might have comparable value as it is easy to calculate and independent of subjective values in an intensive care setting. Thus, it might even be more practical for early patient`s risk stratification in an emergency department, although MELD-XI will certainly not replace well established scoring systems. Optimal cut-off values for MELD-XI scores seem to depend on the primary disease and need to be validated in future prospective studies. In conclusion, the major advantage of the MELD-XI score is that it is easy to calculate, anyhow convenient to determine the risk profile of ICU patients quickly and objectively.

## Supporting Information

S1 Table Shows all relevant data regarding survival and MELD-XI. (XLSX)

S2 Table STROBE checklist. (DOCX)
