# Supplementary material for: Model for End-stage Liver Disease excluding INR (MELD-XI) score in critically ill patients: Easily available and of prognostic relevance
Source: PLoS One. 2017 Feb 2;12(2):e0170987. doi: 10.1371/journal.pone.0170987 (PMC5289507; doi:10.1371/journal.pone.0170987)
Supplement: S2 Table — (DOCX) [file pone.0170987.s002.docx]

STROBE Statement—checklist of items that should be included in reports of observational studies

|  | Item No | Recommendation |
| --- | --- | --- |
| **Title and abstract** | 1 | (*a*) Indicate the study’s design with a commonly used term in the title or the abstract  DONE: A total of 4688 medical patients (66±14 years, 2862 male) admitted to a German ICU between 2004 and 2009 were included and retrospectively investigated |
|  |  | (*b*) Provide in the abstract an informative and balanced summary of what was done and what was found DONE:  The easily calculable MELD-XI score is a robust and reliable tool to predict both intra-ICU and long-term mortality in critically ill medical patients admitted to an ICU. |
| Introduction | | |
| Background/rationale | 2 | Explain the scientific background and rationale for the investigation being reported DONE: Introduction |
| Objectives | 3 | State specific objectives, including any prespecified hypotheses DONE: (…)in the current study we aimed to evaluate MELD-XI in a large collective of critically ill patients admitted to an intensive care unit with respect to prognostic relevance regarding (i) short-term and (ii) long-term mortality. Moreover, we (iii) compared it to existing well-accepted scores (APACHE, SAPS2) and calculated optimal cut-off values. |
| Methods | | |
| Study design | 4 | Present key elements of study design early in the paper DONE: Methods, Study Subjects |
| Setting | 5 | Describe the setting, locations, and relevant dates, including periods of recruitment, exposure, follow-up, and data collection DONE: Study Subjects |
| Participants | 6 | (*a*) *Cohort study*—Give the eligibility criteria, and the sources and methods of selection of participants. Describe methods of follow-up  DONE: Methods, Study Subjects |
|  |  |  |
| Variables | 7 | Clearly define all outcomes, exposures, predictors, potential confounders, and effect modifiers. Give diagnostic criteria, if applicable DONE: Methods |
| Data sources/ measurement | 8* | For each variable of interest, give sources of data and details of methods of assessment (measurement). Describe comparability of assessment methods if there is more than one group DONE: Methods and Results |
| Bias | 9 | Describe any efforts to address potential sources of bias DONE: Limitations |
| Study size | 10 | Explain how the study size was arrived at DONE: Methods |
| Quantitative variables | 11 | Explain how quantitative variables were handled in the analyses. If applicable, describe which groupings were chosen and why DONE: Methods, Statistical analysis |
| Statistical methods | 12 | (*a*) Describe all statistical methods, including those used to control for confounding  DONE: Statistical analysis |
|  |  | (*b*) Describe any methods used to examine subgroups and interactions DONE: Statistical analysis |
|  |  | (*c*) Explain how missing data were addressed DONE: Statistical analysis |
|  |  | (*d*) *Cohort study*—If applicable, explain how loss to follow-up was addressed  DONE: Methods |
|  |  | (*e*) Describe any sensitivity analyses |

Continued on next page

| Results | | |
| --- | --- | --- |
| Participants | 13* | (a) Report numbers of individuals at each stage of study—eg numbers potentially eligible, examined for eligibility, confirmed eligible, included in the study, completing follow-up, and analysed DONE: Methods |
|  |  | (b) Give reasons for non-participation at each stage DONE: Methods |
|  |  | (c) Consider use of a flow diagram |
| Descriptive data | 14* | (a) Give characteristics of study participants (eg demographic, clinical, social) and information on exposures and potential confounders DONE: Methods, Table 1 and Table 2 |
|  |  | (b) Indicate number of participants with missing data for each variable of interest DONE: For survival analysis, Table 4 and MELD-XI in Results |
|  |  | (c) *Cohort study*—Summarise follow-up time (eg, average and total amount) DONE: Methods |
| Outcome data | 15* | *Cohort study*—Report numbers of outcome events or summary measures over time DONE: Table 3 and Table 4 |
|  |  |  |
|  |  |  |
| Main results | 16 | (*a*) Give unadjusted estimates and, if applicable, confounder-adjusted estimates and their precision (eg, 95% confidence interval). Make clear which confounders were adjusted for and why they were included DONE: Statistical analysis, Results |
|  |  | (*b*) Report category boundaries when continuous variables were categorized DONE |
|  |  | (*c*) If relevant, consider translating estimates of relative risk into absolute risk for a meaningful time period |
| Other analyses | 17 | Report other analyses done—eg analyses of subgroups and interactions, and sensitivity analyses  DONE |
| Discussion | | |
| Key results | 18 | Summarise key results with reference to study objectives DONE |
| Limitations | 19 | Discuss limitations of the study, taking into account sources of potential bias or imprecision. Discuss both direction and magnitude of any potential bias DONE |
| Interpretation | 20 | Give a cautious overall interpretation of results considering objectives, limitations, multiplicity of analyses, results from similar studies, and other relevant evidence DONE |
| Generalisability | 21 | Discuss the generalisability (external validity) of the study results DONE |
| Other information | | |
| Funding | 22 | Give the source of funding and the role of the funders for the present study and, if applicable, for the original study on which the present article is based NOT APPLICABLE |

*Give information separately for cases and controls in case-control studies and, if applicable, for exposed and unexposed groups in cohort and cross-sectional studies.

**Note:** An Explanation and Elaboration article discusses each checklist item and gives methodological background and published examples of transparent reporting. The STROBE checklist is best used in conjunction with this article (freely available on the Web sites of PLoS Medicine at http://www.plosmedicine.org/, Annals of Internal Medicine at http://www.annals.org/, and Epidemiology at http://www.epidem.com/). Information on the STROBE Initiative is available at www.strobe-statement.org.
